# A new approach of gene co-expression network inference reveals significant biological processes involved in porcine muscle development in late gestation

**DOI:** 10.1038/s41598-018-28173-8

**Published:** 2018-07-05

**Authors:** M. Marti-Marimon, N. Vialaneix, V. Voillet, M. Yerle-Bouissou, Y. Lahbib-Mansais, L. Liaubet

**Affiliations:** 1 GenPhySE, Université de Toulouse, INRA, ENVT, Castanet Tolosan, France; 2 MIAT, Université de Toulouse, INRA, Castanet Tolosan, France

## Abstract

The integration of genetic information in the cellular and nuclear environments is crucial for deciphering the way in which the genome functions under different physiological conditions. Experimental techniques of 3D nuclear mapping, a high-flow approach such as transcriptomic data analyses, and statistical methods for the development of co-expressed gene networks, can be combined to develop an integrated approach for depicting the regulation of gene expression. Our work focused more specifically on the mechanisms involved in the transcriptional regulation of genes expressed in muscle during late foetal development in pig. The data generated by a transcriptomic analysis carried out on muscle of foetuses from two extreme genetic lines for birth mortality are used to construct networks of differentially expressed and co-regulated genes. We developed an innovative co-expression networking approach coupling, by means of an iterative process, a new statistical method for graph inference with data of gene spatial co-localization (3D DNA FISH) to construct a robust network grouping co-expressed genes. This enabled us to highlight relevant biological processes related to foetal muscle maturity and to discover unexpected gene associations between *IGF2*, *MYH*3 and *DLK1/MEG3* in the nuclear space, genes that are up-regulated at this stage of muscle development.

## Introduction

Cell type diversity in a given organism cannot be explained only by DNA sequences. *Cis-* and *trans*-acting regulatory sequences are not the only determinants of gene expression: other epigenetic mechanisms are also responsible for tissue-specific expression of genes. Indeed, more recently, numerous studies link the genome organization in the nucleus to an additional level of gene expression regulation^1–5^. It is known that in higher eukaryotes, genomes are organized into individual chromosomes that occupy discrete territories in the nucleus^6^, which means that the distribution of the genome is not random. Moreover, interphase chromosome regions often loop out of their chromosome territories^7^, and neighbouring chromosomes can intermingle, resulting in potential functional contacts between regions located on different chromosomes^2–4,8^. There is evidence that long-range interactions between genomic regions contribute to gene expression regulation^2^ and might facilitate the consolidation of co-regulated genes in specialized foci of active RNA polymerase II as well as at nuclear speckles (pre-mRNA processing)^3–5^. These insights give us some clues about the contribution of the spatial genome organization in interphase nuclei to gene expression regulation (for review^9^).

Microscopy approaches such as 3D fluorescent *in situ* hybridization (FISH)^10,11^, enable a global view of what is happening at the level of individual cells. Recently, we focused on this last item to study interchromosomal interactions between co-expressed genes belonging to the Imprinted Gene Network (IGN)^12^. We chose the genomic imprinting model because it can compare, in the same nucleus, the environment of an active allele with an allele maintained as repressed due to its imprinted status. We focused our analysis on *IGF2* because it is involved in pig muscle growth and fat deposition^13,14^, being therefore a major gene of interest in the context of agronomic projects. In humans, IGF2 is well known to be a key element in foetal growth and development^15^. We highlighted associations between the expressed alleles of *IGF2* and *DLK1*/*MEG3* locus (*DLK1* being related to the control of muscle development and regeneration^16^), in foetal muscle and liver cells^17^. These results illustrate the implication in *trans*-interactions of genes associated with quantitative trait loci (QTLs) for growth traits, providing new evidence that genome organization could influence gene expression and phenotypic outcome in livestock species.

In this context, we focused on the study of the muscle maturity process (essential for the survival of piglets) to better understand how interesting phenotypes are elaborated, by combining transcriptome and co-localization data with network modelling. Indeed in pigs, and in general in mammals, one of the most critical period for survival is the perinatal period, and an important determinant of early mortality is maturity, defined as the stage of full development leading to survival at birth^18^. Piglet maturity involves biological processes occurring between the 90^th^ day and the end of gestation, e.g. glycogen accumulation in muscle and liver, as well as maturation of tissues^19,20^. The maturity of skeletal muscles plays an important role in piglet survival at birth because of its involvement in motor functions and thermoregulation. On this subject, we previously performed a microarray analysis of foetal muscle to identify candidate genes for piglet maturity, which revealed genes that were differentially expressed between the 90^th^ and the 110^th^ day of gestation^21^. Using Pearson correlation a relevance gene co-expression network was built from these differentially expressed genes (DEGs) for four gestational ages. The network revealed and confirmed that: (i) genes involved in muscle development were up-regulated at the 90^th^ day of gestation, (ii) at the 110^th^ day, the enriched biological functions were involved in energy metabolism.

An increasing number of studies use gene co-expression networks to deal with large gene expression datasets in order to decipher biological processes^22–24^. Modelling co-expression with network models is useful for providing a global overview of the co-expression relationships between genes and enables a set of genes to be analysed globally with specific network tools. This approach has been found relevant for extracting biological information such as important genes with respect to their centrality in the network structure^25^, densely connected groups of genes^26^ or frequent motifs^27^.

For the study described in this article, we developed a new method for the construction of a co-expression gene network with genes involved in the foetal muscle maturation process, using an original approach coupling a statistical model and observed data in an iterative process to further our understanding of the mechanisms involved in muscle development. More precisely, we combined gene expression data and gene spatial co-location, thus creating a new statistical method for graph inference. Our approach is based on Gaussian Graphical Models (GGMs^28^) that enable the computation of *partial correlations* and fit direct relations better than Pearson-based correlation networks. Such networks have been found to be more efficient for grouping genes with a common function^29^. This enabled us to obtain more reliable networks in which connections between genes were validated iteratively using biological evidence. In practical terms, we performed 3D DNA FISH experiments to test pairwise whether co-expressed genes (connected in the network) were co-localized in the 3D nuclear space.

The study enabled us to obtain a robust gene co-expression network that highlights significant Gene Ontology (GO) terms associated with biological processes related to foetal muscle maturity. In addition, unexpected associations were identified between *MYH3* and the imprinted loci *IGF2* and *DLK1*, which might help elucidate the mechanisms involved in the porcine muscle development process at the end of gestation.

## Results

### Data selection

The 44,368 probes from the expression dataset of the muscle transcriptome study from Voillet *et al*.^21^ were found to correspond to 13,855 unique annotated genes, among which 1,131 unique genes were found to be differentially expressed between the two gestational ages and for the four genotypes characterizing the establishment of piglet maturity. Among them, 359 DEGs (Supplementary Table S1) were selected for being highly correlated with *IGF2*, *DLK1* and *MEG3* (R² ≥ 0.84), also identified as DEG, and were used in all subsequent network inferences (see further details in “Materials and Methods”, the section on “Microarray data description and pre-processing”).

### Network inference iteration and 3D FISH validations

**Figure 1:**
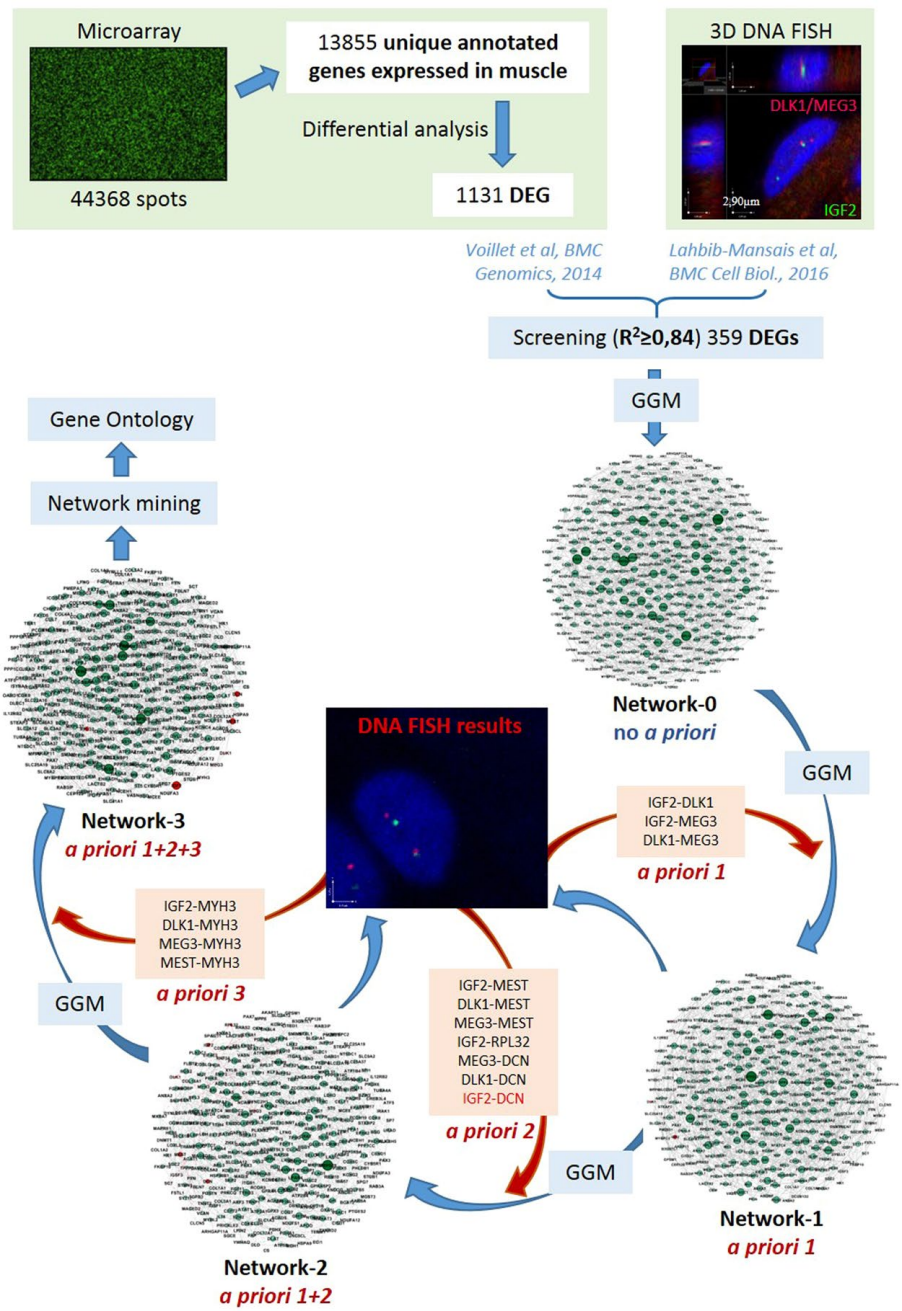
Experimental design. Published data are represented in green squares (microarray data and 3D DNA FISH data), statistical methods are represented in blue (GGM: Gaussian Graphical Models) and new information about spatial localization used for network inference is represented in red.

**Figure 2:**
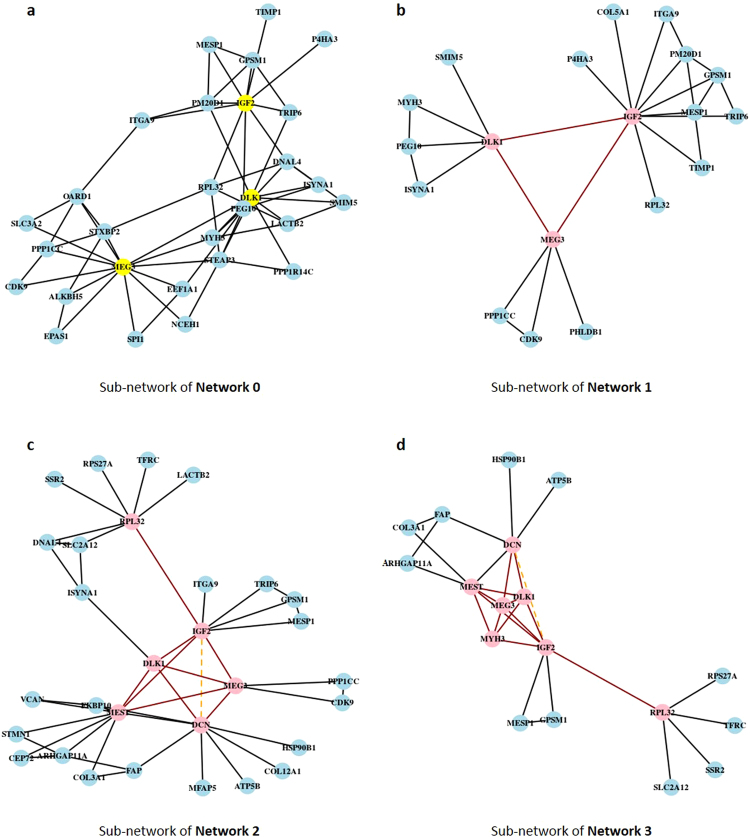
Analysis of gene associations. Pink nodes represent target genes, red edges represent the known associations observed by 3D DNA FISH and the dotted orange edge represents the observed as not associated after 3D FISH validations. Because networks are very dense and contain many genes, a sub-network restricted to the target genes and their direct neighbors is extracted from each network, and presented in this figure. (**a**) Network 0 is inferred without *a priori* information, and restricted to the nodes corresponding to *IGF2*, *DLK1* and *MEG3* (in yellow). To infer Networks 1, 2 and 3, new *a priori* information of spatial localization is introduced for the following pairs of genes: (**b**) *IGF2-DLK1*, *IGF2-MEG3* and *DLK1-MEG3* for Network 1; (**c**) *IGF2-MEST*, (*DLK1/MEG3*)-*MEST*, (*DLK1/MEG3*)-*DCN, RPL32-IGF2, IGF2-DCN* for Network 2; (**d**) *IGF2-MYH3*, *DLK1-MYH3*, *MEG3-MYH3* and *MEST-MYH3* for Network 3.

The whole process involving the data selection, the network inference and the 3D FISH validations is summarized in Fig. 1. Network 0 was inferred with no *a priori* knowledge and contained 2,279 edges for 359 nodes (density: 3.55%). A sub-network extracted around the three target genes is shown in Fig. 2a.

**Figure 3:**
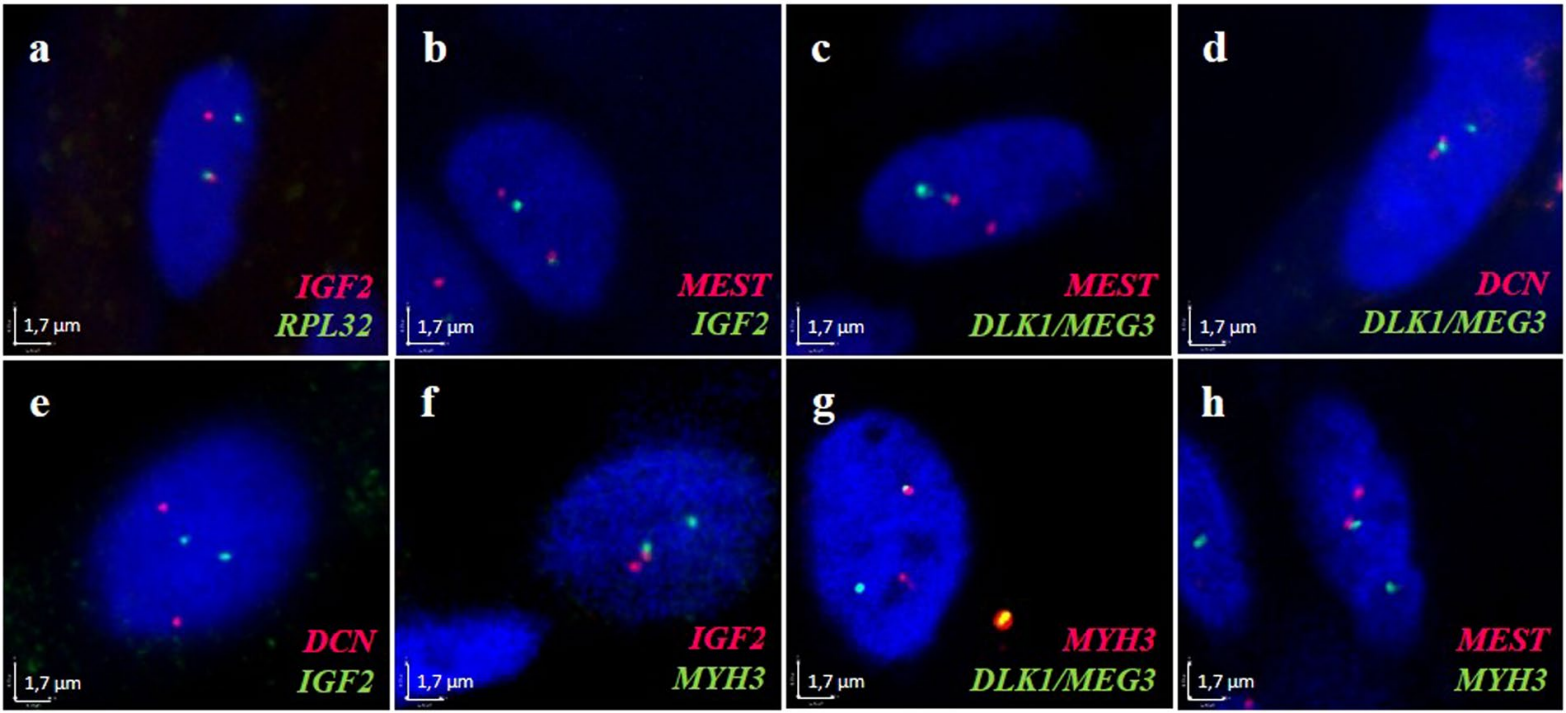
Analysis of gene associations by DNA FISH. Extended focus of 3D image sections from confocal microscopy and overlay of the 3 channels (blue, red and green) were obtained with Volocity v6.0 software (Perkin Elmer). The four signals in the nuclei correspond to the two alleles of each gene. Nuclei are counterstained with DAPI (blue). In all experiments, the percentage of association between genes was higher than 10% except for (e). Scale = 1.7 µm.

Network 1 was built based on the triple co-localization of *IGF2, DLK1 and MEG3* found in our previous study^17^. This *a priori* information was used to reinforce the existence of an edge between the pairs *IGF2-DLK1*, *IGF2-MEG3* and *DLK1-MEG3* in Network 1 (sub-network in Fig. 2b), which contained 2,250 edges (density: 3.50%). In both graphs (Network 0 without *a priori* and Network 1 with *a priori*), we found a direct connection between the genes *IGF2* and *RPL32*. The *IGF2-RPL32* association was thus tested by 3D DNA FISH, because it involved one of our 3 initial target genes (*IGF2, DLK1* and *MEG3*), and because it was also found in the IGN of Varrault *et al*.^12^. The 3D DNA FISH assay revealed that *IGF2* and *RPL32* were associated in 20% of the analysed nuclei (Table 1, Fig. 3a).

**Table 1:** Association percentages of tested gene pairs.

Gene associations	Number of nuclei analysed	Percentage of nuclei with signals			
		Distant (d > 1 µm)	Close (0, 5 < d ≤ 1 µm)	Co-localized (d < 0.5 µm)	Associated (d ≤ 1 µm)
MEST* - IGF2*	100	66	32	2	**34**
MEST* - (DLK1-MEG3)*	90	66	28	6	**34**
DCN - (DLK1-MEG3)*	73	85	15	0	**15**
RPL32 - IGF2*	80	80	16	4	**20**
DCN - IGF2*	98	90	7	3	**10**
IGF2* - MYH3	58	48	43	9	**52**
(DLK1-MEG3)* - MYH3	69	55	38	7	**45**
MEST* - MYH3	103	74	23	3	**26**
ZAR1 - IGF2*	61	92	8	0	**8**
ZAR1 - PRLR	63	92	8	0	**8**

Associated signals (close + co-localized) are considered as those separated by a 3D distance (d) ≤ 1 µm, and are divided into two different classes: “close” signals (0.5 < d ≤ 1 µm), and “co localized” signals (d ≤ 0.5 µm). *Genes imprinted in pig.

Additionally, we used 3D DNA FISH to analyse *MEST* and *DCN* associations with each of the three target genes, because they were also connected in the IGN (Table 1 and Fig. 3b–e).

This new information about spatial co-localization in the nucleus was entered in our model as an *a priori* to build Network 2 (with 2,091 edges and 3.25% of density) (sub-network in Fig. 2c). Specifically, in addition to the three pairs *IGF2-DLK1*, *IGF2-MEG3* and *DLK1-MEG3* given as associated in Network 1, we gave the following pairs of genes as known to be co-localized: *IGF2-MEST* (34% of analysed nuclei presenting an association), (*DLK1/MEG3*)-*MEST* (in 34% of analysed nuclei), (*DLK1/MEG3*)-*DCN* (in 15% of analysed nuclei) and *RPL32-IGF2* (in 20% of analysed nuclei). The pair *IGF2-DCN* was given as not co-localized (with 10% of nuclei presenting an association) (Table 1, Fig. 3b–e). *DLK1* and *MEG3* are two imprinted genes located in the same cluster, and are both present in the same Bacterial Artificial Chromosome (BAC) used for the 3D DNA FISH experiments, because of their proximity on the genomic sequence (Supplementary Table S2). Consequently, we considered *DLK1/MEG3* as a simple locus for all 3D DNA FISH analyses, even though they are considered to be single genes for network inference.

To obtain the last network (Network 3), we used 3D DNA FISH to test for associations involving *MYH3* because it was found to be connected to *DLK1* and *MEG3* in Network 0 and to *DLK1* in Network 1. We found *MYH3* associated with (i) *IGF2* in 52% of the analysed nuclei, (ii) *DLK1/MEG3* in 45% of the analysed nuclei, and (iii) *MEST* in 26% of the analysed nuclei (Table 1, Fig. 3f–h). Thus, in addition to the *a priori* information given in Networks 1 and 2, we gave the following new associations (*IGF2-MYH3*, *DLK1-MYH3*, *MEG3-MYH3* and *MEST-MYH3*) to infer Network 3 (2,091 edges, density = 3.25%) (Sub-network in Fig. 2d).

### Network mining (network structure with key genes)

For each network, two main numerical characteristics (degree and betweenness) were used to detect key genes with respect to the network structure. The degree of a node (in this case, of a gene) is the number of edges afferent to this gene. The betweenness of the node (gene) is the number of shortest paths between pairs of genes in the network that pass through that gene. High-degree genes are connected to many other genes while high-betweenness genes are central and more likely to disconnect the network if removed. We analysed the evolution of the betweenness and degree from Network 0 to Network 3. Supplementary Table S3 shows a subset of 25 genes selected as key genes for the network structure because they showed a high betweenness or a high degree value or both a high betweenness and a high degree, or because they were among genes whose associations tested positive with 3D DNA FISH. Most of the genes presenting the highest betweenness values in Network 0, still kept or increased this numerical characteristic in Network 3 after network inference iterations. However, important changes were observed in some genes. For instance, *AKR7A2, DLK1, EGFR*, *MEG3, MYH3 and RPL32*, showed more than a 40% decrease in betweenness accompanied by a decrease in degree (>25%) when Network 3 was obtained. *DCN* showed a pronounced decrease in its degree while its betweenness was slightly modified. Interestingly, *MEST* and *IGF2* were found to have a mixed profile of betweenness and degree: in Network 3, we observed a 46% loss for *MEST* in gene connections, as compared to Network 0, while its betweenness increased by 160%. Similarly, a 30% loss of connections and a 426% gain in betweenness was observed for *IGF2*.

### Network clustering

To analyse the evolution of the network structure from Network 0 to Network 3, clustering of the genes was performed on each network (for more details, see “Network mining and clustering” in “Materials and Methods” and Supplementary Tables S1 and S4). Four significant clusterings (*p*-value < 0.002) were obtained, one for each network. A total of nine clusters were obtained in Network 0, six in Network 1, eight in Network 2 and six in Network 3. Networks 0 and 3 were analysed in depth to search for any correspondence between clusters (Supplementary Table S5). Four clusters in Network 0 were found to share at least two thirds of their nodes with the corresponding clusters in Network 3. More precisely, 64.1% of the genes in cluster 1, 68.4% in cluster 2, 66% in cluster 3 and 82.4% in cluster 4, were observed in the corresponding clusters of Network 3. The other clusters in Network 0 (clusters 5, 6, 7, 8 and 9) were mainly spread each into two different clusters of Network 3. Additionally, the Normalized Mutual Information (NMI) value was calculated to quantify the similarity between clusterings for pairs of networks (Table 2). Interestingly, we observed that the clustering obtained in Network 0 was the most similar to the clustering obtained in Network 1 (NMI = 0.389). Similarly, the clustering in Network 1 was the most similar to the one obtained in Network 2 (NMI = 0.401), and the clustering in Network 2 was the most similar to the one obtained in Network 3 (NMI = 0.401). This finding suggests that clusterings become more consistent when introducing new biological information in each network inference iteration.

**Table 2:** Normalized mutual information (NMI) between pairs of clusterings.

	Network 0	Network 1	Network 2	Network 3
Network 0	1	0.3893	0.3381	0.3244
Network 1	0.3893	1	0.4007	0.3923
Network 2	0.3381	0.4007	1	0.4152
Network 3	0.3244	0.3923	0.4152	1

NMI measure the similarity between two clusterings. The value is comprised between 0 and 1 and is equal to 1 when the two clusterings are identical.

### Functional enrichment analysis

To test the biological relevance of each cluster in Networks 0 and 3, a functional enrichment analysis was performed for each cluster from both networks. Significant GO terms for Biological Processes (GOBP) were observed in clusters 1 and 2 of Networks 0 and 3, and in clusters 3, 5 and 8 of Network 0 (Table 3 and Supplementary Table S6). Table 3 shows the four clusters presenting the non-redundant GOBP with the smallest False Discovery Rate (FDR). When comparing cluster 1 in Networks 0 and 3, eight common enriched GO terms were observed, mainly involved in extracellular matrix formation, embryonic development, metabolic processes and cellular response to stimulus. Besides, fourteen common enriched GOs were observed in cluster 2 of Networks 0 and 3. These GO terms were mainly involved in cellular respiration, energy metabolism, cellular metabolic processes and metabolism of fatty acids. Additionally, two GO terms were observed only in cluster 2 of Network 3, both involved in the mitochondrial respiratory processes. Interestingly, the smallest FDR were observed in Network 3: (i) for cluster 1 (containing all genes tested by 3D DNA FISH), referring to the “Extracellular structure” term (involving the Decorin gene *(DCN)*; FDR = 1.14e-08); (ii) for cluster 2, referring to the “Generation of precursor metabolites and energy” term (FDR = 1.32e-07) (Table 3).

**Table 3:** Comparison of GOBP in clusters 1 and 2 between Network 0 and Network 3.

GO ID	GOBP Terms	Network 0 - Cluster 1		Network 3 - Cluster 1	
		Genes	FDR	Genes	FDR
43062	Extracellular structure	*POSTN, COL1A1, COL1A2, COL3A1, COL5A1, COL16A1, LAMA4, MFAP5*	5,76E-05	*POSTN, COL1A1, COL1A2,COL3A1, COL5A1, COL5A2, COL16A1*, ***DCN***, *FAP, FBN1, ABI3BP, ANXA2, LAMA4*	**1,14E-08**
71417	Cellular response to organonitrogen compound	*COL1A1, COL1A2,COL3A1, COL5A2, COL16A1, FYN, KLF3, ZFP36L1, HSP90B1*	**6,80E-04**	*COL1A1, COL1A2,COL3A1, COL5A2, COL16A1, DNMT1, FBN1*, ***IGF2***, *HSP90B1*	1,16E-02
45995	Regulation of embryonic development	*COL5A1, COL5A2, FGFR1, LAMA4, LFNG*	2,24E-03	*COL5A1, COL5A2, FGFR1, LAMA4, LFNG*	1,16E-02
71559	Reponse to transforming growth factor beta	*POSTN, COL1A1, COL1A2, COL3A1, FYN, ZFP36L1*	**2,35E-03**	*POSTN, COL1A1, COL1A2,COL3A1, FBN1*	1,24E-01
44236	Multicellular organism metabolic process	*COL1A1, COL1A2,COL3A1, COL5A1, COL5A2*	2,35E-03	*COL1A1, COL1A2,COL3A1, COL5A1, COL5A2, FAP*	3,05E-03
43588	Skin development	*COL1A1, COL1A2, COL3A1, COL5A1, COL5A2, ZFP36L1*	**3,18E-03**	*COL1A1, COL1A2, COL3A1, COL5A1, COL5A2*	1,44E-01
1101	Reponse to acid chemical	*COL1A1, COL1A2,COL3A1, COL5A2, COL16A1, NFATC4*	1,17E-02	*COL1A1, COL1A2,COL3A1, COL5A2, COL16A1, DNMT1, NFATC4*	2,27E-02
1501	Skeletal system development	*POSTN, COL1A1, COL1A2, COL3A1, COL5A2, FGFR1, TMEM119*	1,43E-02	*POSTN, COL1A1, COL1A2, COL3A1, COL5A2, FBN1, FGFR1, ANXA2, TMEM119*, ***IGF2***	3,05E-03
		**Network 0 - Cluster 2**		**Network 3 - Cluster 2**	
72350	Tricarboxylic acid metabolic process	*CS, DLAT, DLD, NNT, MDH1, PDHA1*	3,02E-06	*CS, DLAT, DLD, NNT, MDH1, PDHA1*	2,11E-05
51186	Cofactor metabolic process	*COQ7, DLAT, DLD, NNT, HK1, ACACB, NMNAT3, ACAT1, MDH1, PDHA1, PDHX*	**2,97E-05**	*DLAT, DLD, IBA57, NNT, GPI, ACACB, NMNAT3, MDH1, PDHA1, FLAD1, MCEE*	1,34E-03
72524	Pyridine-containig compound metabolic process	*DLD, NNT, HK1, NMNAT3, MDH1, PDHA1, PDHX*	**1,00E-04**	*DLD, NNT, GPI, NMNAT3, MDH1, PDHA1*	1,11E-02
6631	Fatty acid metabolic process	*CPT1B, ECI1, DLAT, DLD, ACACB, ACADS, ACAT1, PDHA1, PTGES2, PDHX*	**1,00E-04**	*CPT1B, ECI1, DLAT, DLD, FABP3, ACACB, ACADS, PDHA1, ADIPOR2, PTGES2, MCEE*	1,17E-03
6091	Generation of precursor metabolites and energy	*CS, DLAT, DLD, NNT, HK1, MDH1, OXA1L, ATP5B, PDHA1, SLC25A3*	1,09E-04	*CS, DLAT, DLD, NNT, GPI, MDH1, NDUFA3, NDUFB5, NDUFS1, OXA1L, ATP5B, PDHA1, SLC25A3, CISD1, NDUFA12, PYGM*	**1,32E-07**
6090	Pyruvate metabolic process	*DLAT, DLD, HK1, PDHA1, PDHX*	5,42E-03	*DLAT, DLD, GPI, PDHA1, BSG*	2,32E-02
6790	Sulfur compound metabolic process	*VCAN*, ***DCN***, *DLAT, DLD, ACACB, ACAT1, PDHA1, PDHX*	**7,47E-03**	*DLAT, DLD, IBA57, ACACB, PDHA1, MCEE*	4,79E-01
42180	Cellular ketone metabolic process	*COQ7, DLAT, DLD, ACACB, PDHA1, PDHX*	1,46E-02	*DLAT, DLD, FABP3, GPI, ACACB, PDHA1*	8,05E-02
45454	Cell redox homeostasis	*TXNRD2, DLD, NNT, PTGES2*	1,46E-02	*TXNRD2, DLD, NNT, PTGES2*	4,91E-02
44282	Small molecule catabolic process	*CPT1B, ECI1, DLD, HK1, ACACB, ACADS, ACAT1*	1,88E-02	*CPT1B, ECI1, DLD, GPI, ACACB, ACADS, BCAT2, MCEE*	4,51E-02
98656	Anion transmembrane transport	*CLCN5, CPT1B, ACACB, SLC25A3, SLC1A3, VDAC1*	**2,31E-02**	*CPT1B, ACACB, SLC25A3, SLC1A3, VDAC1*	3,77E-01
6081	Cellular aldehyde metabolic process	*DLAT, DLD, PDHA1, PDHX*	2,59E-02	*DLAT, DLD, GPI, PDHA1*	8,73E-02
43648	Dicarboxylic acid metabolic process	*DLD, NMNAT3, MDH1, SLC1A3*	3,13E-02	*DLD, NMNAT3, MDH1, BCAT2, SLC1A3*	2,13E-02
16042	Lipid catabolic process	*CPT1B, ECI1, ACACB, ACADS, ACAT1, NCEH1*	3,65E-02	*CPT1B, ECI1, FABP3, ACACB, ACADS, NCEH1, MCEE*	6,59E-02
10257	NADH dehydrogenase complex assembly			*NDUFA3, NDUFB5, NDUFS1, OXA1L, NDUFA12*	**3,29E-03**
97031	Mitochondrial respiratory chain complex I biogenesis			*NDUFA3, NDUFB5, NDUFS1, OXA1L, NDUFA12*	**3,29E-03**

GO terms enriched in one of the clusters as well as all GO terms associated to one of the three target genes at least (even if not significantly enriched). In bold, the smallest FDR value for a given GOBP term when the difference between the FDR of the two clusters is higher than one order of magnitude. Genes tested by 3D DNA FISH are in underline bold.

These results suggest that our approach to network inference by incorporating *a priori* biological information enables us to obtain relevant GO terms while conserving the functional enriched terms found in the initial network (Network 0). Moreover, we unexpectedly observed that two (*IGF2* and *DCN*) of our seven target genes showed more significant GO terms in Network 3 than in the initial network. Specifically, *IGF2* was observed to be uniquely involved in the “Genetic imprinting” term in cluster 3 of Network 0 (FDR = 3.82e-02), while in cluster 1 of Network 3 it was found to be involved in two new significant GO terms, the one with the smaller FDR being “Skeletal system development” (FDR = 3.05e-03) (Table 3 and Supplementary Table S6). *DCN* was in turn observed to be involved in the “Sulphur compound metabolic process” term (FDR = 7.47e-03) in cluster 2 of Network 0, while in cluster 1 of Network 3 it appeared to be involved in the “Extracellular structure” term presenting the smallest FDR value (1.14e-08) of all clusters. Concerning *MEST*, *MYH3* and *DLK1*, also tested by 3D DNA FISH, even though the observed FDR were higher than 5%, interesting GO terms were observed for these genes in cluster 1 of Network 3 (Supplementary Table S6). For instance, *MEST* was found to be involved in “Mesoderm development”, *MYH3* in “Body morphogenesis”, *DLK1* in “Notch signalling pathway” and *DCN* and *MYH3* were both found to be involved in “Muscle organ development”.

**Figure 4:**
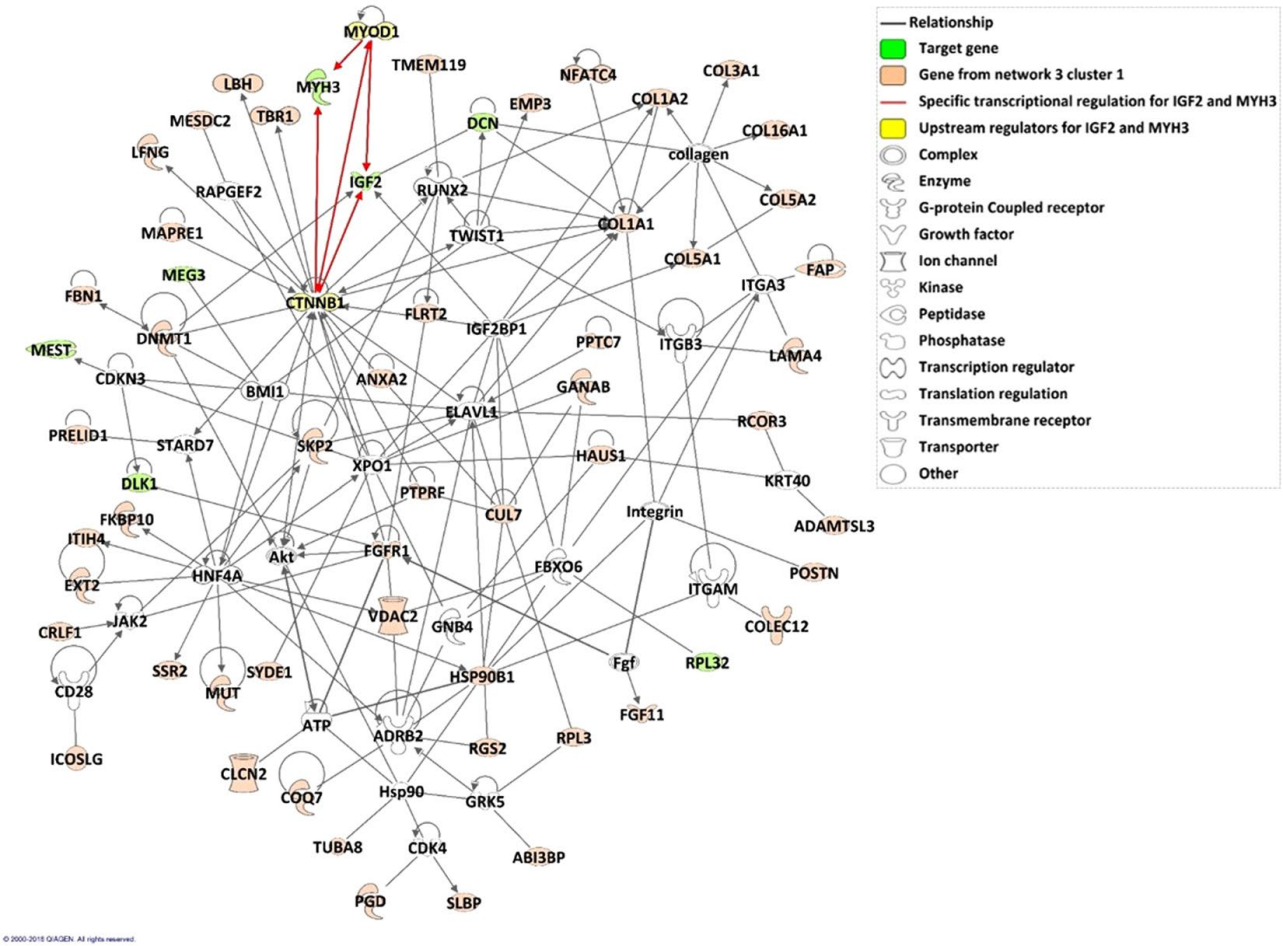
Reconstructed network of genes in cluster 1 of Network 3, based on Ingenuity Pathways Knowledge Base. Nodes are displayed using various shapes that represent the functional class of the gene product. The reconstructed network was generated through the use of Ingenuity Pathway Analysis (IPA) (Ingenuity Systems; QIAGEN, Inc., Valencia, CA, USA).

Another functional analysis was performed with Ingenuity Pathway Analysis (IPA) specifically on cluster 1 of Network 3, which contains the target genes (*IGF2*, *DLK1*, *MEG3*, *RPL32*, *MEST*, *DCN* and *MYH3*). IPA proposed to connecting 49 (82%) out of 60 genes in a network including all target genes except *MEG3* and *MYH3*. *MYH3* was found in a small network with 8 out of 60 genes, and *MEG3* in another small network of only 1 out of 60 genes. Furthermore, *MYOD1* and *CTNNB1* were identified by upstream regulator analysis as potential transcriptional factors for a group of genes including *IGF2* and *MYH3*. As IPA offers the possibility of merging networks (if there are links between nodes in the Ingenuity Pathways Knowledge Base), a reconstructed network was obtained (Fig. 4), and analysed around the target genes. Fourteen genes, among them 7 genes from cluster 1 (including *DCN* and *IGF2*), were observed to be related to “Cell Morphology” (*p*-value = 1.75e-08). *DCN*, *DLK1* and *IGF2* were likewise involved in the “Quantity of cells” function with 31 genes, including 16 genes from cluster 1 (*p*-value = 2.48e-09).

“Morphology of connective tissue cells” with 8 genes (*p*-value = 1.27e-04) included *DLK1* and *MEST*. “Formation of muscle”, with 10 genes (*p*-value = 2.98e-05), involved *IGF2* and *MYH3* together with the two transcription factors *CTNNB1* and *MYOD1* (Supplementary Table S8).

## Discussion

We present here a new approach based on GGM that enables the user to introduce previously acquired biological knowledge to build gene co-expression networks. Since an observed correlation between two genes in the co-expressed gene network does not necessarily mean that these genes are related to a common biological process, we used information of gene nuclear co-localizations to reinforce observed links in the co-expressed gene network. Some studies have shown examples of co-expressed and co-localized genes being implicated in a particular process, e.g. the *Hbb* and *Hba* Klf1-regulated globin genes were found to be co-localized in specialized Klf1-enriched transcription factories of erythroid cells^3^. Others have observed a role of co-expressed and co-localized genes in gene expression regulation, e.g. in the HUVECs endothelial cell line, *SAMD4A*, *TNFAIP2* and *SLC6A5* TNFα-induced genes were hierarchically transcribed when engaged in chromosomal interactions^30^.

In order to determine which pairs of genes would present a reinforced edge in the networks, we performed two negative controls (see “gene-gene associations” in the “Materials and Methods” section). As discussed in our previous study^17^, it can be difficult to define a suitable non-associating control. Sandhu *et al*. established a threshold of 2%^31^, while others used the expected frequency of random co-localization based on the volume of the nucleus and individual gene signals (<1%)^5^. This estimation of random co-localization does not take into account other constraints such as: (1) chromosomes occupy specific territories^4,6^; (2) transcriptionally silent domains reside at the nuclear periphery^32^; (3) chromatin regions are preferentially associated in topological domains (TADs)^33^. Fixing an arbitrary threshold of 10% was a more restrictive way of analysing co-expressed genes that might tend to interact preferentially. Consequently, the pair *IGF2-DCN* was given as not co-localized by enforcing the absence of an edge between both genes.

Testing the nuclear co-localization of *IGF2* and *RPL32* by 3D DNA FISH proved interesting, as this connection concerned an imprinted gene (*IGF2*, involved in muscle growth-related traits^14^) and a ribosomal protein coding gene *RPL32*^34^. This experiment revealed that these genes are associated. Additionally, it was interesting to find co-localized pairs of genes such as *IGF2-MEST*, *(DLK1/MEG3)-MEST*, *(DLK1/MEG3)-DCN*, that were observed to be connected in co-expression networks in other studies^12,23^, even though they were not directly connected by an edge in our network (Network 1) but via intermediary genes. Besides, surprising results showed the highest association we have ever observed between two genes (neither in the present study, nor in previous ones). This association concerns *MYH3* and *IGF2*. *MYH3* plays an important role in foetal muscle development^35,36^, and encodes for the embryonic Myosin Heavy Chain (MYHC) 3 protein. To the best of our knowledge, no previous association between these two genes, whatever its origin (nuclear or functional), has ever been observed, even though the two genes are known to be involved in muscle development^35,37^. To determine the impact of the *a priori* co-localization information introduced to enforce the presence or the absence of an edge, we analysed the evolution from Network 0 to Network 3, first globally (with conserved edges and key genes) and then locally (with network clustering and functional enrichment). The global analyses revealed that 82% of edges in Network 0 were conserved in Network 3 and that the most important genes (with respect to network structure) in Network 0 were among those showing the highest values of betweenness and degree in Network 3. These findings suggest that the introduction of enforced edges is not linked to the appearance of major disturbances in the network structure. However, when focusing on the target genes analysed by 3D DNA FISH, we observed a general decrease in the degree value, meaning that *IGF2*, *DLK1*, *MEG3*, *RPL32*, *MEST*, *DCN* and *MYH3* were less connected with the rest of the other genes in Network 3. Despite this observed isolation concerning genes for which edges were enforced, this effect was not always accompanied by a loss of betweenness. In other words, reinforcing a limited number of edges did not change either the global network structure or the importance of target genes in the final network. In the local analysis, the NMI value revealed that the clusters resembled one another more with each new network inferred. In addition, four out of six clusters in the final network (Network 3) conserved more than 62% of genes in the corresponding clusters of Network 0. This concurred with the results of the functional enrichment analysis, which revealed that the GOs found were conserved between Networks 0 and 3. All these results support the evidence that our approach did not introduce any substantial disturbance. In fact, this iterative process brought substantial improvements; notably, it enabled us to obtain reliable networks in terms of relevant biological information, especially around our target genes. This was supported by the following findings: (1) the biological processes presenting the smallest FDR were found in Network 3, even though one of them involved *DCN*, for which edge estimations were modified by the introduction of *a priori* information; (2) two new significant GO terms related to energy metabolism appeared in cluster 2 of Network 3; (3) two genes (*IGF2* and *DCN*) analysed by 3D DNA FISH were involved in biological processes with smaller FDR in Network 3 than in Network 0. Moreover, *IGF2* was found in an additional GO of Network 3, while only present in one GO of Network 0.

One of the most important goals of the present article was to elucidate the mechanisms that govern porcine skeletal muscle development in late gestation. Many studies have been performed in pig to address this question^21,24,38–41^. In our model, we proposed a final network (Network 3) in which enriched biological functions related to muscle development were observed. These observations were in agreement with the results obtained by Voillet *et al*.^21^. In addition, in the resulting IPA reconstructed network, we highlighted *MYOD1* and *CTNNB1* among the proposed transcription factors because they were especially interesting due to their connection to two important target genes, *IGF2* and *MYH3*. Although *MYOD1* and *CTNNB1* were not present in the 359 genes used for network inference, they were up-regulated at 90 days of gestation in all genotypes (Supplementary Fig. S7)^21^. *MYOD1* encodes for a myogenic factor that regulates skeletal muscle cell differentiation by activating transcription of muscle-specific target genes (for review^42^). *CTNNB1* (β-catenin 1), encodes for a transcriptional co-activator that was found to be required for muscle differentiation in murine myoblasts by interacting directly with MyoD and promoting its binding to the E box elements enhancing its transcriptional activity^43^. The co-expression and nuclear co-localization of *IGF2* and *MYH3* suggest they are each subjected to similar transcriptional regulation by these two transcription factors. The studies of Shang *et al*.^44^ and Ramazzotti *et al*.^45^ are in agreement with this hypothesis. Shang *et al*. revealed that in mesenchymal stromal cells from rats, an ectopic expression of *Ctnnb1* inhibits adipogenetic differentiation and induces the formation of long multinucleated cells expressing myogenic genes, such as *MyoD* and *Myhc*, by promoting the expression of skeletal muscle-specific transcription factors. Ramazzotti *et al*. observed that an overexpression and accumulation of β-catenin in the nuclei of differentiating murine myoblasts results in higher *MyoD* activation and *Myhc* induction. Additionally, *IGF2* was found to be up-regulated in pig during myogenesis and, more precisely, involved in primary and secondary muscle fibre differentiation^41^. Moreover, *Myod* and *Igf2* were observed to be involved in the switch between myogenic and adipose lineages in mouse^46^. In addition, we found *IGF2* indirectly associated with *CTNNB1* (through the intermediary gene *IGF2BP1*) in the reconstructed network. *IGF2BP1* was not used for network inference but was found expressed at the 90^th^ day of gestation (Supplementary Fig. S7)^21^. Indeed, β-catenin was observed to induce *IGF2BP1* in HEK293 cells^47^, which in turn was observed to regulate *IGF2* mRNA subcellular location and translation in neurons (for review^48^). This suggests that in muscle cells, a similar mechanism could possibly be involved for the regulation of *IGF2* via the CTNNB1 transcription factor. Moreover, the long non-coding DNA of *MyoD* (*lncMyoD*), directly activated by MyoD, may negatively regulate *Igf2bp1*-mediated translation of proliferation genes in murine myoblasts^49^. This could explain how MyoD blocks proliferation to create a permissive state of differentiation. Moreover, *DLK1* and *MYOD1* were not connected in the reconstructed network. However, *DLK1* which encodes for a preadipocyte factor that inhibits adipocyte differentiation^50^, might inhibit cell proliferation and enhance cell differentiation by regulating the expression of *MyoD*^16^. Combining all this information with the observed up-regulation at 90 days of gestation of the above-mentioned genes, our results highlight a network of interrelated genes associated with skeletal muscle regulation and that are mainly responsible for inhibition of proliferation and muscle differentiation.

## Conclusion

The innovative approach presented here has proven to be consistent, robust and reliable for the inference of gene co-expression networks in combination with gene nuclear co-localizations. The information generated by the final network brought to light relevant functions involved in the development and maturity of foetal muscle. In this context, the challenge for future studies will be to broaden this approach and render it more powerful by combining co-expression data with information about genome-wide interactions^51,52^ to enforce edges in the network. This study also spotlights interesting gene associations in the three-dimensional nuclear space of muscle cells such as the associations found between *MYH3-IGF2* or *MYH3-*(*DLK1/MEG3*). The three genes are up-regulated in LW at 90 days of gestation and are involved in muscle development. Determining through further functional studies whether and how these genes are co-regulated, will help us to understand the mechanisms involved in the establishment of pig muscle maturity.

## Materials and Methods

### Ethics Statement

All tissues sampled for the experiments were collected on pigs bred for another project (ANR-09-GENM-005-01, 2010–2015). The experiment authorization number for the experimental farm GenESI (Genetics, testing and innovative systems experimental unit) is A-17-661. The procedures performed in this study and the treatment of animals complied with European Union legislation (Directive 2010/63/EU) and French legislation in the Midi-Pyrénées Region of France (Decree 2001-464). The ethical committee of the Midi-Pyrénées Regional Council approved the experimental design (authorization MP/01/01/01/11). All the foetuses used in this study were males and were obtained by caesarean.

### Microarray data description and pre-processing

Expression data were obtained from skeletal muscle for two foetal gestational ages (90 and 110 days of gestation) associated with four foetal genotypes (two extreme breeds for mortality at birth –Large White (LW) and Meishan (MS)– and two reciprocal crosses –MSxLW and LWxMS). The final dataset consisted of 44,368 probes for 61 samples under eight different conditions (four genotypes at two gestational ages). A precise description of the experimental design and data collection can be found in Voillet *et al*.^21^. Normalized expression data (log2-transformed) and sample information are available in NCBI (GEO accession number GSE56301).

Missing values were imputed with k-NN (R package “impute” function, with *k* = *3*). Gene annotation was updated (nblast/NCBI July 2017, Sscrofa10.2) and the 40,847 annotated probes were found to correspond to 13,855 unique genes. For each gene, the probe with the highest average correlation with the other probes associated with the same gene was selected to serve as a representative in further statistical analyses.

### Network inference

Networks were inferred using Gaussian Graphical Models (GGMs^28^) from *n* = 61 samples. From expression data, GGMs build a graph (or network) in which vertices are genes and edges represent the conditional dependency structure between those genes. GGMs are based on the estimation of partial correlations (*i.e*., correlations between two gene expressions when the expression of all the other genes is known). They were preferred over relevance networks^53^ because they improve measurement of direct relations between gene expressions by accounting for the effect of all expression data, and because they were found to be more efficient for grouping together genes with a common function in a previous study^29^.

Since the number of samples was smaller than the number of genes used for network inference, the models were fitted with a sparse penalty^54^ to address the issues of high-dimensional data and edge selection. In addition, as many examples have shown that co-expressed genes occasionally tend to interact preferentially or consolidate in specialized foci of the nuclear environment^2–5^, when *a priori* information about nuclear gene co-localization is available, the latter was included in the model using the approach described in Villa-Vialaneix *et al*.^55^. The details of the method and of the tuning of the different parameters are given in Supplementary Methods online.

### Practical implementation of network inference

The starting point of the analysis was the inference of a network with no *a priori* information about co-localization. Since network inference based on partial correlation can only be performed with a limited number of genes (because of the number of samples) and since the number of unique genes (*p* = 13,855) was too great compared to the number of samples (*n* = 61), we applied two restrictions to the original list. First, we restricted the list to genes that were reported as differentially expressed (DEG)^21^. Secondly, among these DEGs, only those that had an absolute value for their correlation with either *IGF2*, *DLK1* or *MEG3* larger than 0.84 were kept. This final list contained 359 genes, provided in Supplementary Table S1.

### Network inference iteration and 3D FISH validations

Based on network inference results or on genes found to be connected in the IGN of Varrault *et al*.^12^, 3D DNA FISH experiments were performed to check whether pairs of genes of interest were co-localized in the 3D nuclear space. These experiments were conducted in an iterative manner with network inference. More precisely, network inference was performed with the following *a priori* conditions: (1) Network 0: was inferred with no *a priori* information, as a baseline for comparison; (2) Network 1: was inferred using *a priori* information from the triple association found in Lahbib-Mansais *et al*.^17^ by giving the three pairs *IGF2-DLK1, IGF2-MEG3* and *DLK1-MEG3* as known co-localized genes. Network 1 was then used to propose candidate pairs of genes for testing by 3D DNA FISH for Network 2 (*IGF2-RPL32*) and Network 3 (*DLK1-MYH3*); (3) Network 2: in addition to the initial three pairs, Network 2 was inferred using *a priori* information provided by the results of the new 3D DNA FISH experiments by giving the pairs *IGF2-MEST*, *DLK1-MEST*, *MEG3-MEST*, *MEG3-DCN*, *DLK1-DCN*, and *RPL32-IGF2* as known to be co-localized and *IGF2-DCN* as known not to be co-localized; (4) Network 3: in addition to the 10 previous pairs, Network 3 was inferred using *a priori* information provided by the results of new 3D DNA FISH experiments by giving the additional pairs *IGF2-MYH3, DLK1-MYH3 MEG3-MYH3* and *MEST-MYH3* as known co-localized genes.

All simulations were performed with the free statistical software R (https://cran.r-project.org). The inference was performed using our own scripts (available at https://github.com/tuxette/internet3D) and the graphs were displayed and analysed using the R package igraph (Csardi and Nepusz)^56^.

### Network mining and clustering

Nodes of importance to the network structure were obtained by computing the degree and the betweenness centrality measurement for every node. Node clustering was performed by applying the Louvain algorithm^57^, which performs fast approximate optimization of the modularity^58^. All clusterings were found to be significant using the permutation test described in Montastier *et al*.^59^ by generating 500 random networks with the same degree distribution (all clusterings were found to have a modularity larger than that obtained on the 500 random networks, *p*-value < 0.002). Clusters were compared using two methods: first, pairwise contingency tables between clusters were computed. Second, the normalized mutual information (NMI^60^) between pairs of clusterings was obtained. The NMI is a number between 0 and 1 measuring the similarity between two clusterings and is maximum (equal to 1) when the two clusterings are identical.

### Functional analysis of the networks

Functional enrichment analysis based on GO was performed using the web tool Webgestalt (WEB-based GEne SeT AnaLysis Toolkit, http://www.webgestalt.org/option.php) updated on January 27, 2017^61,62^. The web tool uses the Fisher exact test and controls for the number of false positives among the declared significant GOs terms. The False Discovery Rate was used (Benjamini-Hochberg procedure^63^, FDR < 5%). The analysis was performed using the Overrepresentation Enrichment Analysis (ORA) method, selecting non-redundant Biological Processes (BPs). The final network was analysed through the use of Ingenuity Pathway Analysis version 01–12 (updated on March 31^st^, 2018). Ingenuity Pathway Analysis (IPA, Ingenuity Systems; QIAGEN, Inc., Valencia, CA, USA, https://analysis.ingenuity.com/pa) contains a large bibliographic database (Ingenuity Pathways Knowledge Base) with various molecular relationships already identified between two genes (protein-protein interaction, ligand-receptor regulation, enzymatic modification, transcriptional expression regulation, etc.). The obtained network is a graphic representation of the molecular relationships between molecules. All edges are supported by at least one reference from the literature, or from canonical information stored in the Ingenuity Pathways Knowledge Base. The obtained networks were improved for representation using Path Designer. Nodes are displayed using various shapes that represent the functional class of the gene product. The Functional Analysis identified the biological functions, the canonical pathways and the upstream regulators that were the most relevant to the dataset. Molecules from the dataset that were associated with biological functions, canonical pathways or upstream regulators in the Ingenuity Knowledge Base were considered for the analysis. Fisher’s exact test was used to calculate a right-tailed *p*-value determining the probability that each function and pathway assigned to that dataset is due to chance alone. The networks proposed by IPA were cleaned (some nodes/genes were discarded) in order to keep only the genes necessary to connect the co-expressed genes. The three first networks were merged and regulation information was added to highlight transcription factors that could explain unexpected gene co-expression and nuclear co-localization (e.g. *MYH3* and *IGF2*; Supplementary Table S8).

### Tissue preparation

Foetal muscle tissue was obtained from the *Longuissimus dorsi* muscle of 90-day gestation ♀MSxLW♂ pig and prepared as described in^17^ with slight modifications. When needed, stored muscle fibre packets were permeabilised for 8 min in cytoskeleton extraction buffer (100 mM NaCl, 300 mM sucrose, 3 mM MgCl2, 10 mM PIPES pH 6.8) containing 0.5% Triton X-100 and then fixed in cold 4% paraformaldehyde for 5 min. After washing in cold PBS, muscle packets were manually dilacerated directly on Superfrost glass slides (CML, Nemours, France) to isolate individual fibres, and air-dried before adding DNA probes for *in situ* hybridization.

### Probes construction

Bacterial artificial clones (BACs) containing genes were isolated from porcine BAC libraries (available at the Biological Resources Center-GADIE, INRA, Jouy-en-Josas, France http://abridge.inra.fr/) using specific primers designed with Primer3 software (http://primer3.sourceforge.net/) (Supplementary Table S2). For multiple-label experiments, approximately 120 ng of each BAC DNA was random-priming labelled directly by incorporation of dUTP Alexa Fluor (488 or 568) or indirectly with Biotin-6-dUTP detected by immuno-FISH (Bioprime DNA labelling kit, Invitrogen, Cergy Pontoise, France). Chromosomal localizations of all BAC probes were controlled by 2D DNA FISH on porcine metaphases prepared from lymphocytes according to standard protocols^64^.

*IGF2* had been localized previously on SSC2p17, *DLK1/MEG3* on SSC7q26 and *ZAR1* on SSC8q11-12^17^. In this study, additional genes were localized on pig metaphases: *MYH3* on SSC12q, *MEST* on SSC18, *RPL32* on SSC13q24-33, *DCN* on SSC5qter, and *PRLR* on SSC16 (Supplementary Table S2).

### 3D DNA-FISH on interphase nuclei

3D DNA FISH experiments were conducted using specific probes to label each gene with a different colour as described in^17^ with slight modifications. Probes were resuspended in hybridization buffer (50% formamide, 10% dextran sulphate, 2 mg/ml BSA, 2× SSC) at a final concentration of 110 ng/µl. Nuclear DNA and probes were simultaneously denatured at 74 °C for 7 min and then incubated overnight at 37 °C in a wet atmosphere (DAKO hybridizer). Washes were then performed with gentle agitation, first twice in 2× SSC at room temperature (RT) for 8 min, then twice for 3 min in 2× SSC, 50% formamide pH 7.0 at 40 °C, and finally twice for 15 min in 2× SSC, then in PBS at RT. When a biotin-labelled probe was used, biotins were detected by incubating the slides with streptavidin-Alexa 568 or 488 for 1 hour at RT.

### Confocal microscopy and image analyses

Image stacks were captured at different depths with a Leica TCSSP2 confocal microscope (Leica Instruments, Heidelberg, Germany) equipped with an oil immersion objective (plan achromatic 63× N.A. = 1.4). The Z-stacks (around 60 confocal planes per capture) were acquired at 1024 × 1024 pixels per frame using an 8-bit pixel depth for each channel at a constant voxel size of 0.077 × 0.077 × 0.284 μm. Images were analysed with specific software for measuring the 3D distances (centre-to-centre) between signals (genes) (NEMO^65^) as described in^17^. Euclidean distances were computed with respect to the x, y and z resolutions. Given the resolution on the z axis, at least three pixels corresponding to 0.852 μm (0.284 × 3) were required for a high resolution between two separate signals; consequently, 1 μm was chosen as the upper cut-off for associated signals.

### Gene-gene associations

In all 3D DNA FISH experiments, nuclei were only analysed when 4 signals (corresponding to the 2 alleles of each gene) were present. “Associated” signals were considered to be those separated by a distance (d) ≤ 1 µm, and were divided into two different classes: “close” signals (0.5 < d ≤ 1 µm), and “co-localized” signals (d ≤ 0.5 µm). The great majority of associations concerned uniquely one allele from each gene. To establish the threshold for distinguishing between associated and non-associated genes, two 3D DNA FISH experiments were performed as negative controls: first, between two genes (*ZAR1* and *PRLR*) located on different chromosomes and expressed at a very low level in muscle cells^21^, second, between *IGF2* (highly expressed) and *ZAR1* (low expression)^17^. In both cases, the two genes were found to be associated in only 8% of the analysed nuclei. Considering this value as a sporadic association between loci not expected to be associated, a 10% value was arbitrarily chosen to distinguish between associated and non-associated genes.

### Data availability

The data sets supporting the results of this article are available in the NCBI’s Gene Expression Omnibus repository, and are available through GEO Series accession number GSE56301.

## Electronic supplementary material

Supplementary information

Supplementary_Table_S1

Supplementary_Table_S2

Supplementary_Table_S3

Supplementary_Table_S4

Supplementary_Table_S5

Supplementary_Table_S6

Supplementary_Fig._S7

Supplementary_Table_S8
