# Supplementary material for: A new approach of gene co-expression network inference reveals significant biological processes involved in porcine muscle development in late gestation
Source: Sci Rep. 2018 Jul 5;8:10150. doi: 10.1038/s41598-018-28173-8 (PMC6033925; doi:10.1038/s41598-018-28173-8)
Supplement: Supplementary file 1 — Supplementary information [file 41598_2018_28173_MOESM1_ESM.pdf]

Description of the model used for network inference

Supplementary file for the article "A new approach of gene co-expression network inference reveals highly significant biological processes in pig muscle involved in the etablishment of maturity"

This file describes the model used for network inference and puts it in perspective with other approach found in the litterature. It also explains the choices made for the different hyper-parameters of the method.

1 Network inference

Networks were inferred using Gaussian graphical models (GGM; Edwards, 1995) from $n = 61$ samples at gestational age 90. From expression data, GGM build a graph (or network) in which vertices are genes and edges represent a strong relationship between the gene expressions. GGM are based on the estimation of partial correlations (*i.e.*, correlations between two gene expressions knowing the expression of all the other genes). They were preferred over relevance networks (Butte and Kohane, 2000) because they better measure direct relations between gene expressions by accounting for the effect of all expression data and because they were found more efficient to group genes with a common function in a previous study (Villa-Vialaneix et al., 2013).

More precisely, if $X = (X_1, \ldots, X_p)$ denotes the random variables corresponding to the expression of p genes, GGM supposes that X follows a Gaussian distribution $\mathcal{N}(0, \Sigma)$ and aims at estimating

$$\text{Cor}\left(X_j, X_{j'} | (X_k)_{k \neq j,j'}\right)$$

for every pair (j, j') in $\{1, \ldots, p\}$. A graph is obtained from these estimation by putting an edge between nodes corresponding to the variables X_j and $X_{j'}$ when this partial correlation is different from 0. It can be shown that estimating partial correlations is also equivalent to estimating $\beta_{jj'}$ in the following linear models:

$$X_j = \sum_{j'=1,\ldots,p, j' \neq j} \beta_{jj'} X_{j'}$$

and more precisely that

$$\beta_{jj'} \neq 0 \qquad \Leftrightarrow \qquad \text{Cor}\left(X_j, X_{j'} | (X_k)_{k \neq j,j'}\right) \neq 0.$$

When the number of samples is smaller than the number of genes used for network inference (which is generally the case and which was the case for our problem), the estimation of the partial correlation or of the equivalent linear models are ill-posed problems. This issue is frequently addressed by adding a sparse (L_1) penalty to the maximum likelihood (ML) problems induced by the linear regression formulation: this is the Graphical Lasso (GLasso) (Friedman, Hastie, and Tibshirani, 2008). This method allows to simultaneously estimates the coefficients $\beta_{jj'}$ and to perform variable (here edge) selection among the possible candidates since Lasso penalty yields to provide sparse solution in which many coefficients $\beta_{jj'}$ are set to 0 by the maximization of the penalized likelihood.

Similarly to that approach, we used a model that included a sparse penalty (for edge selection) combined with two L_2 (smooth) penalties aiming at incorporating *a priori* information into the inference similarly to what is proposed in Villa-Vialaneix et al., 2014. More precisely, this led to the minimization over $\beta_{jj'}$ (for j

and j' varying from 1 to p) of

$$\underbrace{\frac{1}{2}\beta_j^\top \widehat{\Sigma}_{\backslash j \backslash j}\beta_j + \beta_j^\top \widehat{\Sigma}_{j\backslash j}}_{\text{pseudo maximum likelihood}} + \lambda \underbrace{\|\beta_j\|_1}_{L_1 \text{ (sparse) penalty}} + \mu \underbrace{\sum_{(k,j)\in E_1} (\beta_{jk}-1)^2}_{L_2 \text{ (smooth) penalty for co-localized edges}} + \mu \underbrace{\sum_{(k,j)\in E_2} (\beta_{jk}-0)^2}_{L_2 \text{ (smooth) penalty for non co-localized edges}} \quad (1)$$

in which $\widehat{\Sigma}$ is the empirical estimates of Σ, $\widehat{\Sigma}_{\backslash j \backslash j}$ is the same matrix deprived from row and column j, $\widehat{\Sigma}_{j\backslash j}$ is row j of the empirical covariance matrix deprived from entry j, E_1 is the list of known co-localized genes and E_2 is the list of genes known not to be co-localized. λ and μ are two positive hyper-parameters that respectively control the sparsity of the solution and its conformity to *a priori* co-localization of information.

The idea behind the model of Equation (1) is that edge estimation must be enforced for pairs of genes that are known to be co-localized whereas the absence of an edge must be enforced for pairs of genes that are known not to be co-localized.

2 Practical implementation of network inference

The same method, based on a bootstrapping scheme than the one described in (Villa-Vialaneix et al., 2014) was used to perform the inference while ensuring the robustness of the estimation: $B = 100$ bootstrap samples were drawn from the original dataset. Inference (*i.e.*, the minimization, for all $j = 1, \ldots, p$, of Equation (1)) was performed for every bootstrap sample and a fixed value of μ. The inference was performed for the complete set of values for λ along the regularization path (Friedman, Hastie, and Tibshirani, 2010). The value of λ that ensured at least T_1 edges in the network was kept (and T_1 was set to 20% of the number of pairs of nodes in the network). Only edges that appear in, at least, $T_2 = 15$ bootstrap samples were included in the final network.

Finally, μ was set to the minimum value such that all *a priori* information were recovered, which led to $\mu = 0.2$ in Network 2, $\mu = 0.3$ in Network 3, $\mu = 0.4$ in Network 3. All simulations were performed with the free statistical software R (R Core Team, 2017) (`https://cran.r-project.org`). The inference was performed using our own scripts (available at `https://github.com/tuxette/internet3D`) and the graphs were displayed and analyzed using the R package **igraph** (Csardi and Nepusz, 2006).

Bibliography

Butte, A. and I. Kohane (2000). “Mutual information relevance networks: functional genomic clustering using pairwise entropy measurements”. In: *Proceedings of the Pacific Symposium on Biocomputing*, pp. 418–429. DOI: 10.1142/9789814447331_0040.

Csardi, G. and T. Nepusz (2006). “The igraph software package for complex network research”. In: *InterJournal* Complex Systems. URL: http://igraph.sf.net.

Edwards, D. (1995). *Introduction to Graphical Modelling*. New York, USA: Springer.

Friedman, J., T. Hastie, and R. Tibshirani (2008). “Sparse inverse covariance estimation with the graphical lasso”. In: *Biostatistics* 9.3, pp. 432–441. DOI: 10.1093/biostatistics/kxm045.

– (2010). “Regularization paths for generalized linear models via coordinate descent”. In: *Journal of Statistical Software* 33.1, pp. 1–22.

R Core Team (2017). *R: A Language and Environment for Statistical Computing*. R Foundation for Statistical Computing. Vienna, Austria. URL: http://www.R-project.org.

Villa-Vialaneix, N. et al. (2013). “The structure of a gene co-expression network reveals biological functions underlying eQTLs”. In: *PLoS ONE* 8.4, e60045. DOI: 10.1371/journal.pone.0060045.

Villa-Vialaneix, N. et al. (2014). “Inferring networks from multiple samples with concensus LASSO”. In: *Quality Technology and Quantitative Management* 11.1, pp. 39–60. URL: http://www.cc.nctu.edu.tw/~qtqm/qtqmpapers/2014V11N1/2014V11N1_F3.pdf.
