# Supplementary figures and images for: A new approach of gene co-expression network inference reveals significant biological processes involved in porcine muscle development in late gestation
Source: Sci Rep. 2018 Jul 5;8:10150. doi: 10.1038/s41598-018-28173-8 (PMC6033925; doi:10.1038/s41598-018-28173-8)

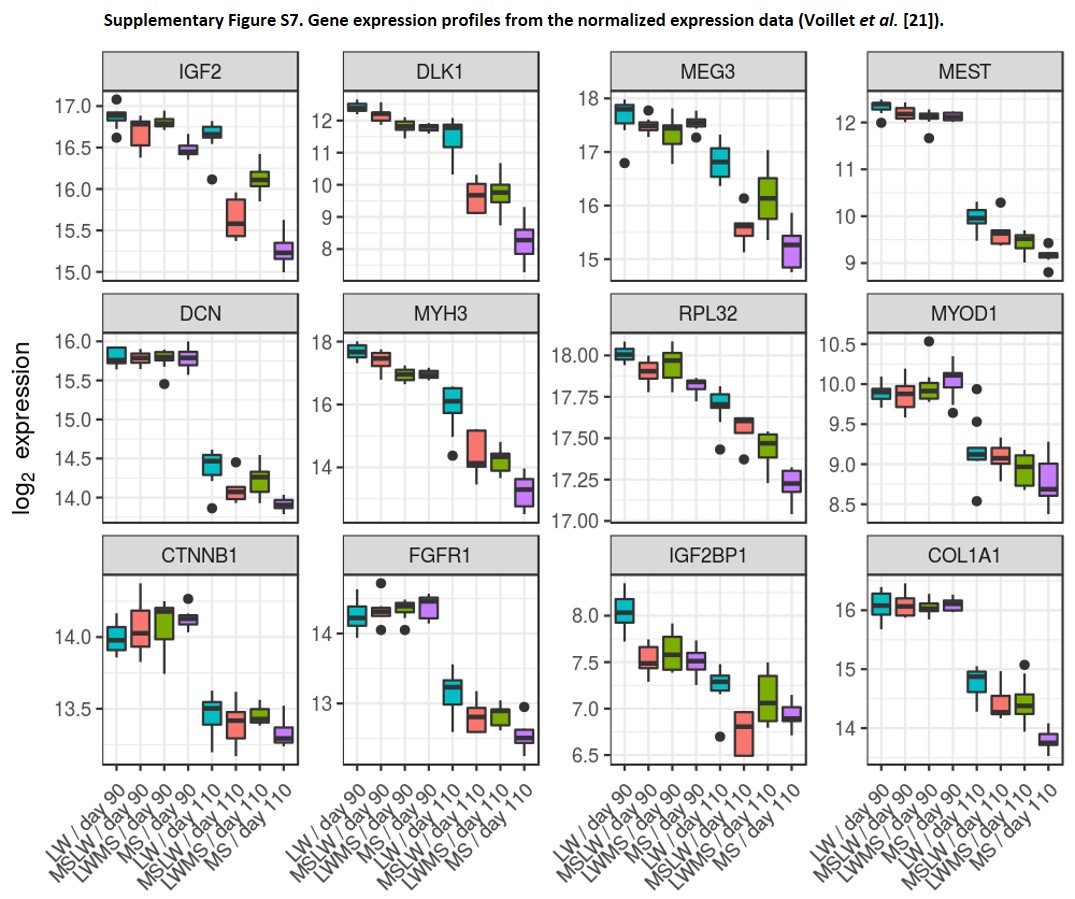

Supplement: Supplementary file 8 — Supplementary_Fig._S7 [file 41598_2018_28173_MOESM8_ESM.jpg]
